# SASC: Secure and Authentication-Based Sensor Cloud Architecture for Intelligent Internet of Things

**DOI:** 10.3390/s20092468

**Published:** 2020-04-27

**Authors:** Khalid Haseeb, Ahmad Almogren, Ikram Ud Din, Naveed Islam, Ayman Altameem

**Affiliations:** 1 Department of Computer Science, Islamia College Peshawar, Peshawar 25000, Pakistan; khalid.haseeb@icp.edu.pk (K.H.); naveed.islam@icp.edu.pk (N.I.); 2 Chair of Cyber Security, Department of Computer Science, College of Computer and Information Sciences, King Saud University, Riyadh 11633, Saudi Arabia; 3 Department of Information Technology, The University of Haripur, Haripur 22620, Pakistan; ikramuddin205@yahoo.com; 4 Department of Natural and Engineering Sciences, College of Applied Studies and Community Services, King Saud University, Riyadh 11543, Saudi Arabia; aaltameem@ksu.edu.sa

**Keywords:** wireless sensor network, Internet of Things, sensor-cloud, unsupervised learning, node’s security

## Abstract

Nowadays, the integration of Wireless Sensor Networks (WSN) and the Internet of Things (IoT) provides a great concern for the research community for enabling advanced services. An IoT network may comprise a large number of heterogeneous smart devices for gathering and forwarding huge data. Such diverse networks raise several research questions, such as processing, storage, and management of massive data. Furthermore, IoT devices have restricted constraints and expose to a variety of malicious network attacks. This paper presents a Secure Sensor Cloud Architecture (SASC) for IoT applications to improve network scalability with efficient data processing and security. The proposed architecture comprises two main phases. Firstly, network nodes are grouped using unsupervised machine learning and exploit weighted-based centroid vectors for the development of intelligent systems. Secondly, the proposed architecture makes the use of sensor-cloud infrastructure for boundless storage and consistent service delivery. Furthermore, the sensor-cloud infrastructure is protected against malicious nodes by using a mathematically unbreakable one-time pad (OTP) encryption scheme to provide data security. To evaluate the performance of the proposed architecture, different simulation experiments are conducted using Network Simulator (NS3). It has been observed through experimental results that the proposed architecture outperforms other state-of-the-art approaches in terms of network lifetime, packet drop ratio, energy consumption, and transmission overhead.

## 1. Introduction

In the last few years, wireless sensor networks (WSNs) gained a lot of research interest from the research community due to its dynamic nature and wide range of applications [1,2]. A WSN comprises tiny smart devices called micro-sensors with limited memory, storage, processing, and battery resources. There are different kinds of sensors used based on applications such as measuring pressure, temperature, humidity, and mobility [3,4]. All sensed information is further forwarded to the base station (BS) via the appropriate forwarding node called the cluster head. As the network size and nodes’ density increases, the network scalability with data security is most of the challenging tasks for WSNs [5,6,7,8,9].

In recent years, many solutions have been presented to improve the routing and energy efficiency for limited constraints networks, however, most of them overlooked data authentication and security in the presence of malicious nodes. The dynamic nature of Internet of Things (IoT) systems allow an enormous number of heterogeneous physical and virtual objects and are interconnected via the Internet [10,11,12,13,14,15]. The paradigm of IoT enables these objects to communicate with each other in a distributed manner, however, the communication provides a lack of trust in data security [16]. Moreover, IoT networks typically need to be optimized for energy consumption and load-balancing schemes and have to be developed to route the IoT system for a longer life with reliable data delivery. Therefore, developing an efficient and intelligent IoT communication system to ensure data security with network consistency is a demanding challenge [17,18,19,20,21,22].

To transmit data packets towards the BS, the presented solution is categorized into chain based, tree-based, and cluster-based routing protocols. Chain and tree-based routing protocols forward data packets to the root node using the next-hop selection method usually based on distance factor. However, such methods have a high latency ratio and therefore they are not appropriate for large scale networks [23,24,25]. On the other hand, cluster-based protocols are mainly used in high-density scenarios and improve network performance in terms of lifetime and throughput [26,27,28]. In formulated clusters, ordinary nodes sense the targeting environment and forward information to associated cluster heads. Afterward, the cluster heads forward the gathered data towards the BS via a single or multi-hop transmission model. Additionally, the sensor-cloud has become a popular infrastructure due to the limited constraints on the sensor nodes, massive data processing, and data storage. However, cluster heads are operated independently and are therefore exposed to innumerable security threats in the presence of malicious nodes. Although different security routing solutions have been proposed by researchers for IoT based WSNs [18,29,30], they require some high computational mathematical and validation functions, which result in degrading network performance in terms of communication and processing overheads. Furthermore, most of the proposed solutions overlooked node authentication in the presence of malicious threats and may lead to compromised data security [31,32]. Therefore, a secure sensor-cloud based architecture with node-level authentication is needed for real-time applications, aiming toward efficient data processing and data protection against malicious nodes.

This article presents a secure sensor-cloud architecture for IoT based WSN to provide data security and easy to scale with efficient network performance. The proposed architecture’s scope is the deployment of IoT-based WSN in the environment of smart cities. Numerous IoT sensors, identified with unique tags, are distributed in a smart city scenario to sense, gather, and forward data via intermediate gateways or cluster heads towards the BS. The BS is further connected with the cloud server using wireless communication technologies, such as WIFI, 4G, 5G, etc. After receiving the data from gateways or cluster heads, the BS uploads the data related to smart cities on the cloud server via the Internet. The proposed architecture integrates the deployment of IoT-based WSN with cloud infrastructure, therefore, the technologies of smart cities communicate, transfer, and analyze important data to maintain the urban operations. Additionally, due to the limited constraints of IoT sensors, they may be disposed to failure that may slow down the data processing in smart cities. Moreover, malicious or compromised entities can lead to disruption in the data sensing and transmission, which results in critical information that may be lost. Such a situation degrades various operations and services for both the community and city infrastructure. Hence, the deployment of IoT-based WSN in the context of smart cities requires a more robust and secure communication solution to improve the functionalities of an urban area network. The proposed SASC architecture provides significant improvements over existing solutions in terms of higher level of data privacy, integrity, and robust authentication. Firstly, the SASC divides network nodes into various clusters based on dynamic pivotal positioning. The clustering of nodes allows a resourceful load distribution inside each cluster and improves network lifetime. Secondly, to cope with efficient data storage and processing capabilities under restricted constraint nodes, integrating WSNs with cloud infrastructure is adopted. In the end, the proposed SASC gives a secure and authentic algorithm for data protection and node validation in an insecure and unreliable communication environment. The proposed security algorithm requires the least computational time and space capabilities with ease to implement. The aforesaid contributions of the proposed SASC offer a remarkable impact on constraint devices under untrustworthiness and dynamic environment. Additionally, the proposed algorithm provides the node level authentication process to identify malicious nodes and to generate networks more consistent with efficient data delivery performance. The rest of the paper is organized as follows. Section 2 presents the related technology and problem finding of this work. Section 3 introduces SASC with its algorithms and design. The simulation model and numerical results of SASC in comparison with other solutions are discussed in Section 4. Finally, Section 5 concludes the paper.

## 2. Related Work

Recently, smart devices called sensor nodes provided an opportunity to design and maintain the network architecture for information gathering and forwarding. Sensor nodes are mostly dispersed randomly in a self-configured mode for various applications such as fire detection, smart cities, smart home, healthcare, and agriculture [33,34,35]. Since sensor nodes have limited constraints in terms of processing, storage, battery, and transmission capabilities, they might be compromised in unreliable and complex IoT systems. Moreover, the field of artificial intelligence (AI) grown by developments in machine learning (ML) more explicitly in constraint networks showing significance in an area of IoT systems [36,37]. Thus, developing an energy-efficient and intelligent network infrastructure with effective data security is the main research challenge for IoT devices [38,39,40].

In cloud computing [41,42,43], end-users get infrastructure from service providers for storing and processing of data. Due to huge data collection and limited resources of sensor nodes, the cloud infrastructure is integrated into WSNs for strengthening network performance, i.e., computational processing and data storage. In the sensor-cloud architecture, sensor nodes capture required data from the monitoring area and store it on the cloud for processing and analyzing purposes, which is then sent towards the requested end-users or clients. The sensor-cloud infrastructure reduces the overheads on low power sensing nodes and improves network management [44,45,46,47]. The storage of big data and their processing is one of the demanding applications for any cloud infrastructure. However, most of the proposed solutions overlook the security and authentication aspects of the network among cluster heads, BS, and cloud servers. This may disclose the data privacy to malicious nodes and may compromise the network performance [48,49,50].

Clustering solutions [51,52,53] provide improvements in energy efficiency, network scalability, and data delivery performances. However, most of these solutions do not consider secure data forwarding with node-level authentication in the presence of malicious nodes. In the cluster formation phase, the cluster head performs a significant role in data collection and transmission. Under unreliable environments, the cluster head verification with secure data transmission is a major research challenge that can compromise the capabilities of the network. Low Energy Adaptive Clustering Hierarchy (LEACH) is the standard and first dynamic protocol [54], which aims to divide the sensor field into various clusters. However, the formulated clusters are non-uniformly distributed concerning load balancing and lack of secure data transmission. Furthermore, the role of cluster heads is shifted based on a fixed epoch that rapidly executes the process of the re-clustering phase. Authors in [55] proposed LEACH based security routing protocol for WSNs using Exclusion Basis Systems (EBS) and µTESLA. The proposed solution generates and distributes keys based on EBS and guarantees the security of keys by using µTESLA. However, the cluster formation phase is still based on a random manner and leads to uneven energy consumption among sensor nodes. In addition, the security key is frequently updated in each data transmission round.

In [56], the authors proposed an energy-efficient and QoS-aware routing protocol for wireless sensor networks based on a smart grid to achieve reliable data transmission. Furthermore, the proposed solution consists of a BMO-based routing algorithm for uniformly sized energy consumption between the nodes. However, the proposed solution overlooked the data security in terms of data confidentiality, authenticity, and integrity under the presence of network threats. As a result, the transmitting information may be compromised. Authors in [57] proposed SecLEACH that aims to improve the performance of cluster-based solutions by incorporating some functionalities of the data level security. Initially, the BS shares a set of keys among sensor nodes where these keys are drawn from a large key pool. In SecLEACH, cluster heads are selected based on random numbers and nodes join a particular cluster head through the strength of a received signal. Each cluster head determines the message authentication code (MAC) and sends the computed value along with aggregated data towards the BS. Nevertheless, the SecLEACH protocol generates clusters in the same traditional fashion and consumes additional energy in re-clustering. Moreover, the proposed solution overlooked the network threats for data manipulation and source and destination authentication in the data routing.

Authors in [58] proposed an energy-efficient and trust aware routing protocol for Mobile Ad-hoc Networks (MANETs). The rationale behind the presented solution is to offer the trust-based method to solve the issue of node misbehavior. Along with the trust value, each node also measures its rate of energy consumption for data gathering and forwarding. A special table, referred to as Get-Trust, is created and maintained by all nodes, which aims to determine the trust level of their neighbors. However, each node needs extra computational and energy power to compute the trust value of each neighbor. As a result, the proposed solution disturbs the network lifetime. Besides, the proposed solution does not consider node authentication in data forwarding and it lacks optimal and reliable routing decisions. Moreover, a Secure and Energy-efficient Multi path Routing (SEER) protocol [59] is presented which utilizes energy resources in a balanced manner for the network performance. Each node maintains a routing table and determines multi path routing toward end-points. The proposed solution also makes use of residual energy and is exploited by the BS to determine the status of energy in the constructed routing path based on sending and receiving data packets. Nevertheless, SEER consumes extra network costs in the development of multi-path routing. Furthermore, the proposed solution overlooks data security in terms of integrity, privacym and node-level authentication, which results in compromised nodes and network reliability. A secure and authentication protocol in WSN is proposed in [60], which tries to improve data security with minimal communication overheads. The proposed solution uses mutual authentication protocol by using a timestamp and generates a unique session key for the new data transmission phase. The proposed solution needs light communication and computation load, however, it is vulnerable to different attacks. Additionally, it does not consider energy-efficient and optimal routing decisions. To add more, the generation of session keys for all sessions requires additional network overhead and energy consumption.

In [61], the authors proposed a secure knowledge and cluster-based intrusion detection mechanism for smart WSNs. The proposed solution depends on the knowledge base, which is stored and maintained on the BS. The knowledge base stores all events that are triggered by sensor nodes. The proposed solution divides the network field into clusters and each cluster has one cluster head. The cluster head records the behavior of all its members in the format of a unique event. Moreover, all events are forwarded towards the BS and some operations are performed by the BS to compute the load on each cluster head. The proposed solution improves energy efficiency in the network field, however, it imposes an extra computational and storage overload on the part of sensors. Moreover, the optimal decision of the selection of cluster heads is overlooked. The authors in [62] proposed an efficient on-demand latency guaranteed interactive model for sensor-cloud, which aims to reduce the latency rate and energy consumption. The proposed model performs complicated functions on the cloud and light-weight processes are executed on low powered nodes. Moreover, the proposed model presents the aggregation function to reduce the application requests for sensor-cloud. However, the proposed model does not consider optimal and reliable policies for data routing and incurs additional network overheads.

Based on the literature review, it is seen that sensor-cloud is used in various applications such as healthcare, military, smart cities and environmental and monitoring, etc. Due to the unreliable and dynamic infrastructure of such applications, energy efficiency, data security, and node-level authentication are the most demanding challenges. It is observed that most of the proposed solutions do not consider the limitations of low powered sensors while developing a sensor-cloud solution. Although some of the existing sensor-cloud solutions decrease the overhead of network nodes, they lack the optimal data aggregation and routing decision. On the other hand, the knowledge-based security models improve energy efficiency and network lifetime, but these solutions offer data reliability with the additional cost of computation and energy consumption on sensor nodes due to events initiation and management. Furthermore, most of the proposed sensor-cloud solutions do not focus on the measurement of data security and node-level authentication in the presence of malicious threats, which result in compromised network trustworthiness. Therefore, it is concluded from the aforesaid solutions that the sensor-cloud infrastructure is suitable for large-scale network regions to increase network scalability with the addition of proper data management. However, the constraints of low powered sensor nodes should be taken into consideration while developing a solution. Therefore, the main contribution of the proposed solution is to develop a secure and authentic sensor-cloud architecture for the improvement of data gathering and energy efficiency. Moreover, the proposed architecture offers lightweight cryptosystems to estimate data security in terms of confidentiality, integrity, and authentication. Besides, with the integration of cloud to sensor networks, the proposed architecture optimizes computational overheads on sensor nodes with minimum energy consumption.

## 3. The Proposed Secure and Authentic Sensor Cloud Architecture

**Figure 1:**
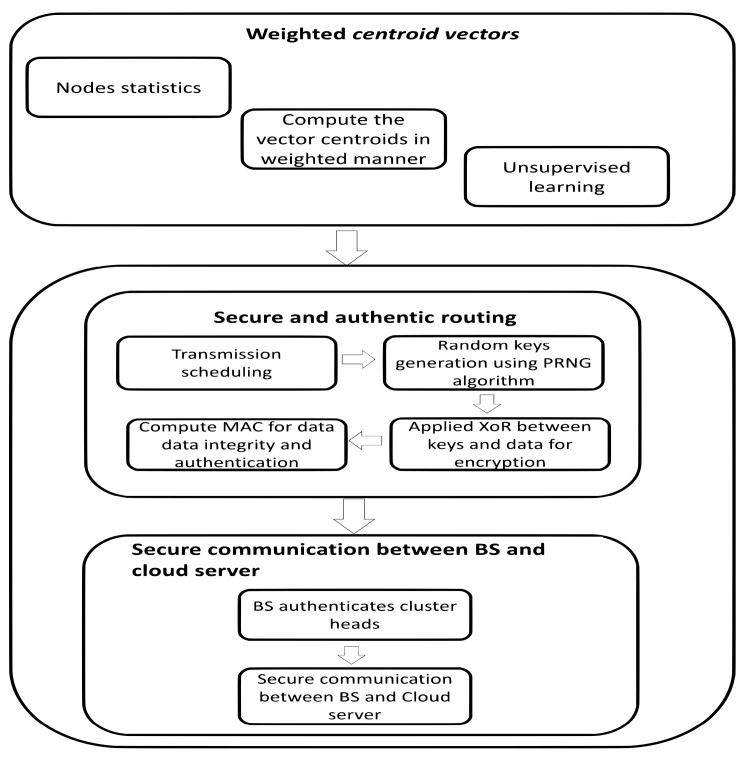
Block diagram of Secure Sensor Cloud Architecture (SASC) Architecture.

This section presents a brief introduction of the proposed secure sensor cloud architecture of IoT based on WSNs. The detail of its algorithms is to be argued in the subsequent subsections. In the first algorithm, network nodes are divided into centroid-based regions and various clusters are represented based on the nodes locality. Unlike other solutions that compute centroid values for the consideration of position factor, our proposed algorithm makes use of both distance and energy factors in weighted means to determine the centroid vectors. Accordingly, quantifiable analysis is being exploited based on the network status. Afterward, the generated clusters are self-organized by using computed centroid vectors and an appropriate cluster head is selected within each cluster to gather and forward sensors’ data towards the BS. In the second algorithm, the sensed data is stored on cloud infrastructure for further processing and retrieving purposes, which results in decreased computational overhead and improved network lifetime. Furthermore, to secure sensors’ data that is stored on cloud infrastructure against malicious threats, the proposed algorithm gives a lightweight security scheme based on the OTP mechanism. The proposed security scheme exploits exclusive-OR (XOR) bitwise operation by the integration of both data bits and random secret keys. The random secret keys are made using a pseudorandom number generator (PRNG) algorithm [63,64], which produces a sequence of bits that are random and never be reused. Due to this mechanism, malicious nodes cannot be able to detect the patterns of key bits. Accordingly, due to the randomness of secret keys, the computed OTP requires the least workout and perfectly secures data transmissions against mischievous entities. Moreover, the proposed solution ensures data integrity and authentication based on message authentication code (MAC) among cluster heads and BS. The block diagram of the SASC is depicted in Figure 1.

### 3.1. Centroid-Based Cluster Formation Algorithm

In the beginning, the initiation component is executed to construct the neighborhood tables and the adjustment of initial routing paths. The BS floods its identity (ID) and location loc(x,y) information in the sensor field. Upon receiving the flooded message, the adjacent neighboring nodes to the BS store the information in their local tables, increment the packet counter and forward the received information to their neighbors. The same practice is followed by all enduring nodes until they have the required information of their neighbors. Afterward, the network nodes send their own and neighbors position information to the BS on the constructed initial routing paths. On receiving, the BS measures the distance of each node from their neighbors and the BS itself in the sensor field. Subsequently, the computed distance information is sent back by the BS to particular nodes. Each node incorporates the received distance calculation in its local table. The main aim of the initialization component is to exchange messages among nodes and BS to have global information of the entire network field.

Basically, in mathematical and engineering applications, the centroid is an arithmetic mean position of the points in a given space S. Unlike other solutions, the proposed SASC makes use of both energy and distance factors to compute the centroid vector. In traditional solutions, the position and weight of nodes do not change during network operations. Thus, the computation of centroids only in the use of position factor is useless. Hence, in robust and dynamic environments, SASC incorporates an energy aspect of nodes along with their distance information in a weighted manner to compute the function f(n) by following Equation (1).

(1) f(n)=α. e(n) +β. c(x,y)

In Equation (1), the contribution of both weighting parameters, i.e., α and β, must be equal to 100% such that α + β = 1. e(n) is the arithmetic mean of residual energy of node n within a precise space S. Let ($n_{1},n_{2}$, $n_{3}$,..., $n_{k})$ is the set of nodes within a particular space $S_{i}\;$, a centroid c(X,Y), which is like a virtual node, can be determined based on positions of the nodes. The c(X,Y) location is computed by taking the mean of the x-coordinates; $X=\frac{1}{n}\overset{n}{\underset{i=1}{\sum }}\left(x_{i}^{2}\right)$, and the mean of the y-coordinates; $Y=\frac{1}{n}\overset{n}{\underset{i=1}{\sum }}\left(y_{i}^{2}\right)$.

In practice, the BS is a more powerful node and has no constraints in terms of resources as compared to other sensor nodes. Based on the position of nodes, the BS computes k number of centroids ($c_{1},c_{2}\;$, $\;c_{3}\;$,..., $c_{k})$ and sends back the information to network nodes. In the proposed SASC, an unsupervised machine learning system is performed by using a k-means algorithm [65] that computes the nearest center of cluster based on computed f(n) . Accordingly, a set of nodes $n_{i}$ that is nearest to j-th centroid of the cluster are grouped to a particular region. This unsupervised machine learning system executes the same procedure continues until all nodes are divided into a unique region. Next, a node that is neighboring to the computed values of f(n) within each region is assigned the role of initial cluster head. All the selected cluster heads broadcast their status with their ID to members and schedule their data transmission slots using time division multiple access Moreover, the role of the cluster head is rotated based on network conditions, such that whenever the selected cluster head drops its energy level to the specified threshold, the next nearest node towards the f(n) value is selected as the new cluster head. Likewise, the newly selected cluster head adjusts the TDMA schedule and broadcasts its information inside a particular region only.

### 3.2. Cloud-Based Data Security Algorithm

In this algorithm, cloud infrastructure is integrated with sensor networks to support the processing of network management and efficient deployment of resources. Although there are several benefits of sensor cloud such as network scalability, computing, and data storage, security is mostly open research issue under the presence of malicious threats and a variety of attacks may be possible. In such a paradigm, the traditional solutions of sensor networks are not appropriate and realistic for cloud infrastructure [66,67]. The proposed algorithm copes with data security in terms of confidentiality, integrity, and authentication based on light-weight cryptosystems. The proposed algorithm prevents malicious nodes from disclosure and tampering of network data. The data security between the BS ($B_{i}\;$) and the cloud server is achieved through the use of an asymmetric based encryption technique. In this technique, the BS and cloud server generate a pair of keys ($k_{p}$ and $k_{u}\;$), whereas $k_{p}$ and $k_{u}$ represents the public and private keys, respectively, where the control of private keys is limited to the cloud server and BS. The generated public keys are used for data encryption and they are shared via a publicly accessible directory that is created on the cloud server. Moreover, the stored public keys on a cloud server are associated with the ID of generated systems. On the other hand, the private keys are not distributed because they are needed to be kept secret. Therefore, they do not leave the system on which it was produced. Similarly, all nodes generate their pair of private-public keys only once and store the public keys along with their IDs on the publicly reachable directory, which is made on the cloud server. In the proposed architecture, the generation of private-public keys is based on the public key cryptosystem of RSA [68]. In the RSA cryptosystem, the key generation is the most important step, where two distinct keys are generated. In this process, two primes p and q are chosen which are kept a secret, and their product  n=p∗q is compute. In the next step, λ(n) is computed, where λ is Carmichael’s totient function with λ(n) = lcm(p−1,q−1). Furthermore, an integer e coprime to λ(n) is chosen which satisfies the two condition, i.e., 1<e<  λ(n) and gcd(e,λ(n)) . Finally, determine d ≡ $e^{-1}\left(mod\;\lambda \left(n\right)\;\right)\;$, where d is the modular multiplicative inverse of e. Thus, the generated public keys are (e,n) and the private keys are (d,n). The encryption of the data ‘D’ from the BS towards the cloud server is accomplished by computing Equation (2).

(2) $$E\equiv \;D^{e}\left(mod\;n\right)$$

The cloud server can decrypt the encrypted data E using Equation (3).

(3) $$D\equiv E^{d}\left(mod\;n\right)$$

For authenticating the cluster heads ($U_{i}$) in the network, the MAC technique is used with each data packet transmitted between the cluster heads and BS. A cluster head $U_{i}$ integrates the data ‘D’ with the private key to generate a short MAC or digital signature. Upon receiving the data ‘D’, the cluster head $U_{j}$ verifies the MAC or digital signature using the corresponding public key of cluster head $U_{i}\;$. For transmitting data $D_{U_{i}}$ from the cluster heads to the BS, the data $D_{U_{i}}$ is XORed with the key $k_{i}$ to produce a MAC or signature AD of the data, as given in Equation (4).

(4) $$AD=D_{U_{i}}⊕k_{i}$$

(5) $$ND=AD+D_{U_{i}}$$

The ND is transmitted from the BS towards the cloud server by encrypting it through the public key generated using the RSA cryptosystem, as given by Equation (2). Upon receiving the encrypted data, the cloud server first decrypts the data using its private key $k_{u}\;$.

Moreover, the private-public key is the combination of well-defined security techniques and offers prevention from impersonation attacks. Both the public and private keys are mathematically related but not the same and provide robust authentication against impersonation attacks. Furthermore, only the public key is globally known via a publicly accessible directory while the private key is to keep secret. The robust authentication of the cluster heads is performed by the BS, which separates the original data $D_{U_{i}}$ and the appended data AD . The original data $D_{U_{i}}\;\;$ is XORed with the corresponding cluster head’s public key $k_{i}$ to produce the MAC, which is matched against the value of appended data AD . If the match is true, the BS acknowledges the verification and authentication process of the cluster head $U_{i}$ and proceeds the transmission and reception activities, else, the BS ignores the data from the cluster heads $U_{i}$ and stops further communications.

## 4. Network Assumptions and Model

In this section, the performance of the proposed SASC architecture is evaluated and compared with secure and authentication protocols, i.e., SEER and SecLEACH. The proposed SASC offers an energy-efficient and data gathering for traditional applications, e.g., WSN, and also presents various kinds of intelligence and secure data routing through IoT sensors. In the simulation setup, we deploy randomly 100 to 500 sensors in the squared sized observing area. The number of malicious nodes is fixed to 10 and dispersed randomly. The malicious nodes broadcast the false route reactions and forwarded the data packets towards unauthorized nodes or can drop the data packets. Public keys are generally known to all sensors via a publicly accessible directory that is made on the cloud server, however, private keys do not need to distribute and therefore they cannot be compromised. All nodes except the BS are limited constraints in terms of memory, storage, processing, and battery power. The transmission power of all the nodes is fixed to 20 m. In the beginning, all nodes have a uniform energy resource of 5J. The numerical results of the proposed SASC architecture are measured against other algorithms in terms of network lifetime, packet drop ratio, energy consumption, average end to end delay, and transmission overhead. Table 1 illustrates the simulation parameters that are used for the computation of numerical results.

## 5. Numerical Results

This section provides results of the proposed scheme in comparison with other two popular methods, i.e., secure and authentication protocol, SEER and SecLEACH, concerning network lifetime, packet drop ratio, energy consumption, end-to-end delay, and transmission overhead.

### 5.1. Network Lifetime

**Figure 2:**
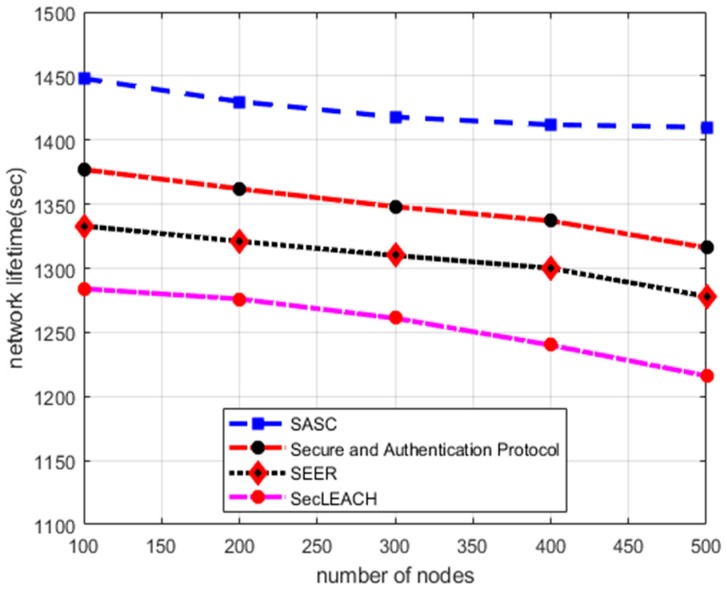
Network lifetime in a varying number of nodes.

**Figure 3:**
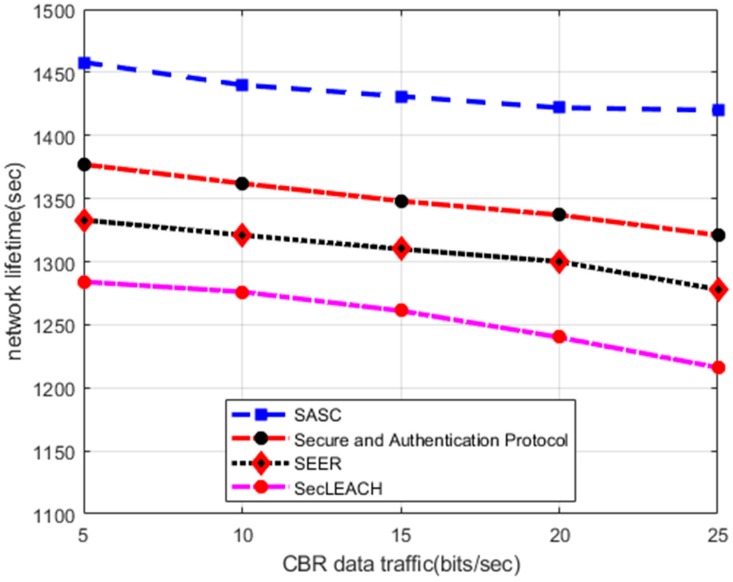
Network lifetime in varying CBR data traffic.

In this section, the numerical results of the proposed SASC architecture with other solutions perform in terms of network lifetime. Figure 2 illustrates that the experimental results of SASC improve network lifetime by an average of 13% in comparison with the existing works under a varying number of nodes. Similarly, on the other hand, the experimental results in Figure 3 demonstrate the improvement of SASC by an average of 12% in the comparison of existing solutions under varying constant bit rate (CBR) data traffic. Such improvements are due to that SASC generates clusters based on nodes locality and balances the energy consumption among sensor nodes. Unlike other solutions that divide the sensor nodes into non-optimized clusters without considering the condition of the nodes and randomly selecting the cluster head, SASC presents an optimal way for cluster formation based on centroid vectors. Moreover, the existing solutions offer data security without considering the constraints of low powered sensor nodes. While the proposed SASC architecture is more simplified in terms of communication overheads and leads to improved network lifetime. Under heavy network load, the existing solutions increase frequent re-transmissions of data packets and route breakages thereby result in a compromised network lifetime.

### 5.2. Packet Drop Ratio

**Figure 4:**
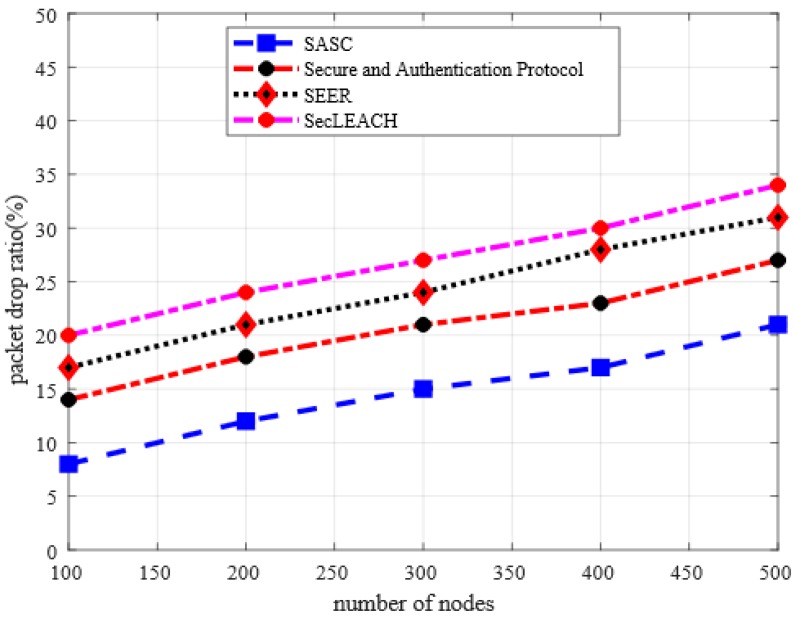
Packet drop ratio in a varying number of nodes.

**Figure 5:**
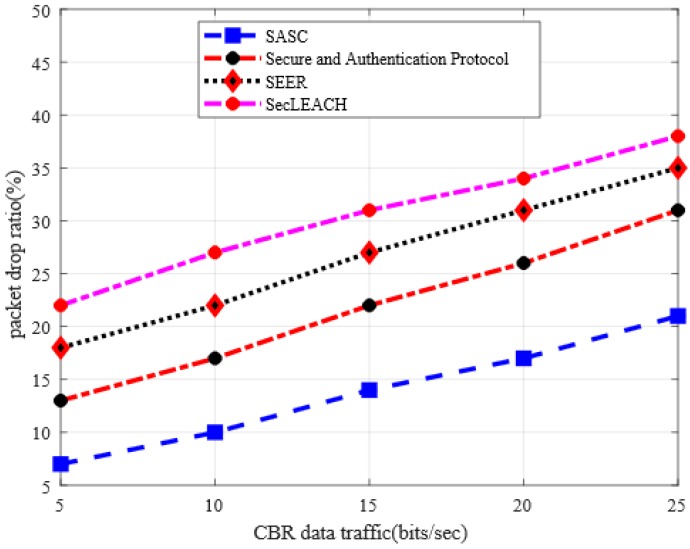
Packet drop ratio in varying CBR data traffic.

This section presents the analysis of the packet drop ratio between SASC and other existing solutions. In Figure 4, the experiment results illustrated that proposed SASC gives better results in terms of packet drop ratio by an average of 37% in the comparison of the existing solution under a varying number of nodes. Similarly, Figure 5 has also proven improved performance of SASC concerning packet drop ratio by an average of 46% in the comparison of other solutions. This is due to the fact that the existing solutions lack the capabilities to detect the congestion quantity on data links because of a large number of nodes and route request packets. In addtion, the existing solutions consume unnecessary energy in data protection and integrity, which reduce the lifetime of routing paths and lead to increased packet drop ratio. On the other hand, SASC decreases the ratio of packet drop due to its lightweight security and data integrity mechanisms under the presence of malicious nodes.

### 5.3. Energy Consumption

**Figure 6:**
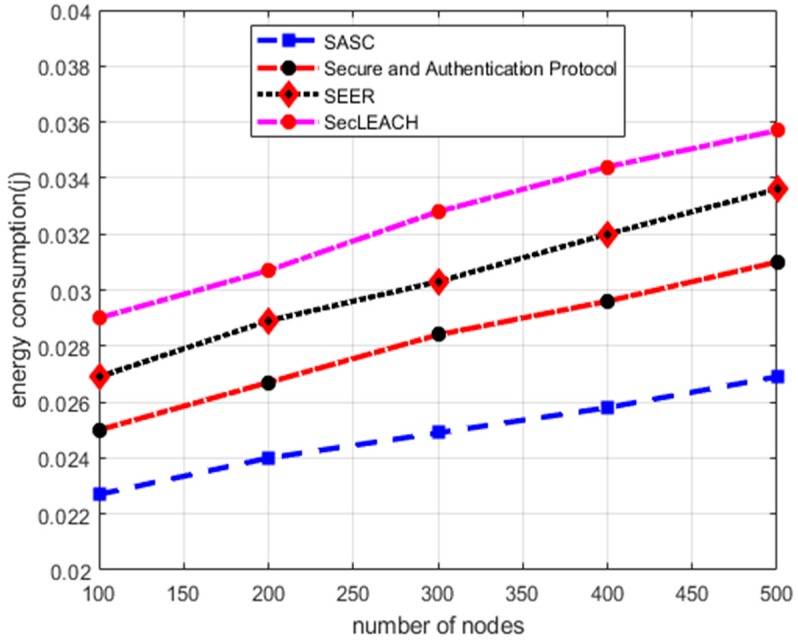
Energy consumption in a varying number of nodes.

**Figure 7:**
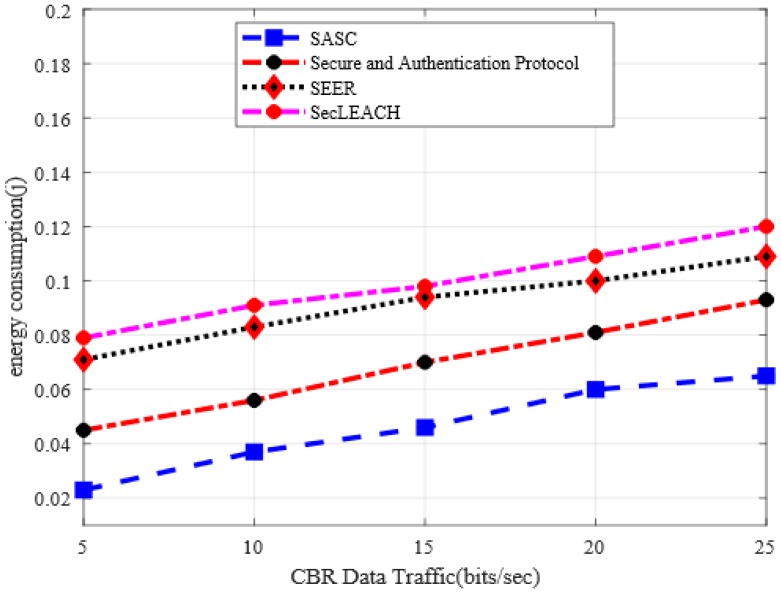
Energy consumption in varying CBR data traffic.

Figure 6 depicts the performance evaluation of SASC architecture with other solutions in terms of energy consumption under a varying number of nodes. Based on numerical results, it is observed that SASC decreases energy consumption by an average of 19% as compared to other solutions. On the other hand, the experimental results in Figure 7 demonstrate the improvement of energy consumption between SASC architecture and the existing solution. And the numerical analysis shows that SASC improves energy consumption by an average of 45% under varying CBR data traffics. The existing solutions cause additional overheads due to frequent route re-discoveries under the presence of malicious nodes and lead to unnecessary energy consumption. Moreover, the cluster heads are rotated on a fixed interval with considering the network status, such mechanisms deplete energy consumption between sensors in an unbalanced manner. The design of SASC architecture focuses on consistent and energy-efficient mechanisms for data forwarding and reducing needless energy consumption over the network field. Moreover, SASC helps to balance a load of energy consumption on the network nodes while forwarding the data on secure and authentic routing paths in the presence of potential security attacks. Additionally, the private-public keys are generated only once by each node and keep the public keys on the publicly accessible directory for global sharing, which greatly reduces the energy consumption in the process of keys management with nominal computational overheads. Moreover, to forward data packets from the cluster heads towards the BS, the proposed architecture exploits a light-weight XOR function between the data and key to produce a MAC or signature, which requires a nominal computational overhead.

### 5.4. Average End-to-End Delay

**Figure 8:**
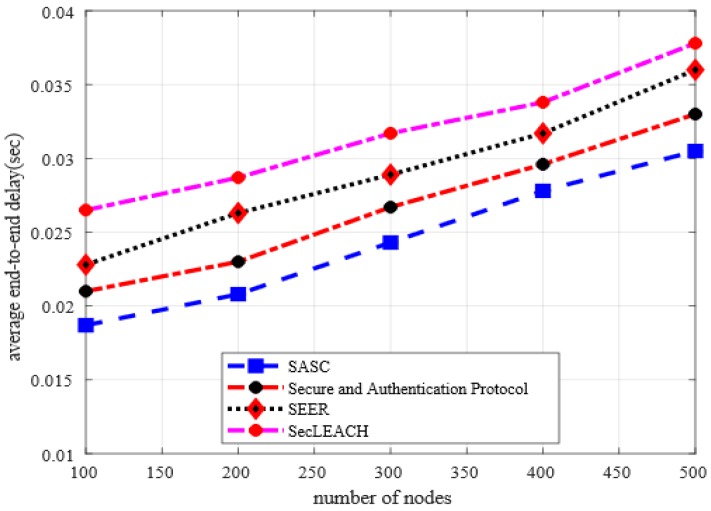
Average end-to-end delay in a varying number of nodes.

**Figure 9:**
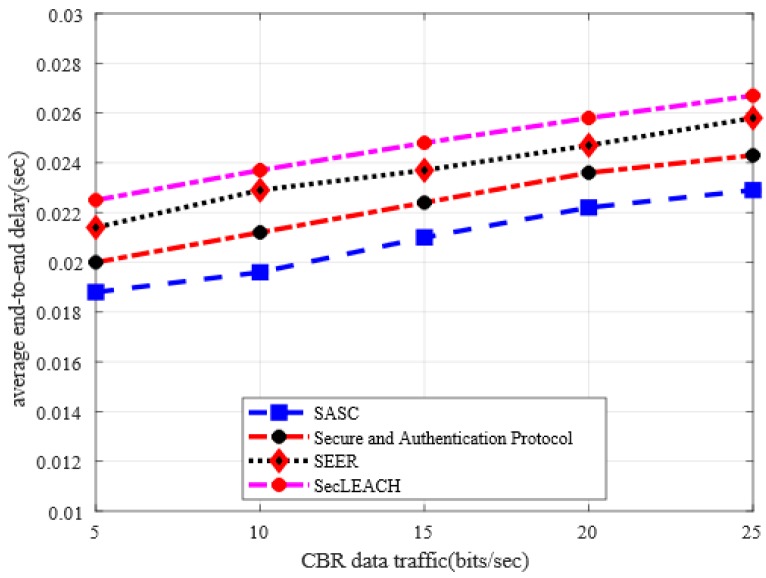
Average end-to-end delay in varying CBR data traffic.

Figure 8 illustrates the behavior of SASC architecture with other solutions in terms of end-to-end delay under a varying number of nodes. The numerical analysis shows that the proposed SASC architecture significantly reduces the rate of end-to-end delay by an average of 16% as compared to the existing solution. Similarly, the numerical analysis in Figure 9 illustrates the improvement for end-to-end delay of SASC by an average of 11% in the comparison of existing work under varying CBR data traffic. Under heavy network traffic and increasing number of nodes, the existing solutions grow the chances of data re-transmissions and incur network disconnections. In addition, due to the unreliable and non-optimal selection of cluster heads, the existing solutions incur a frequent route re-discoveries packet that increases the network delay. The proposed SASC architecture performs data routing on more secure and authentic routes concerning integrity and reliability, which results in decreasing the chances of route failures and data disruption. Besides, once secure data forwarders deplete their energy level in proposed SASC architecture, they evaluate network status and formulate an up-to-date and more energy-efficient routing path to achieve reliable data transmission.

### 5.5. Transmission Overhead

**Figure 10:**
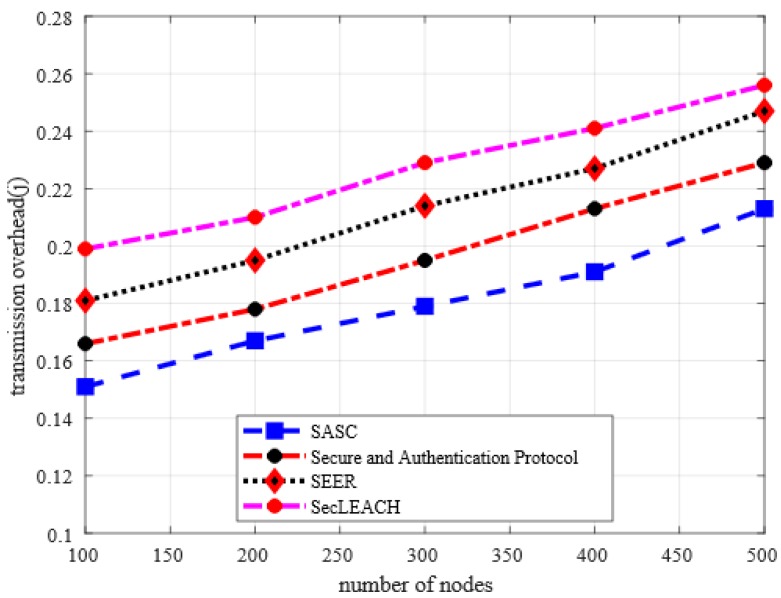
Transmission overhead in a varying number of nodes.

**Figure 11:**
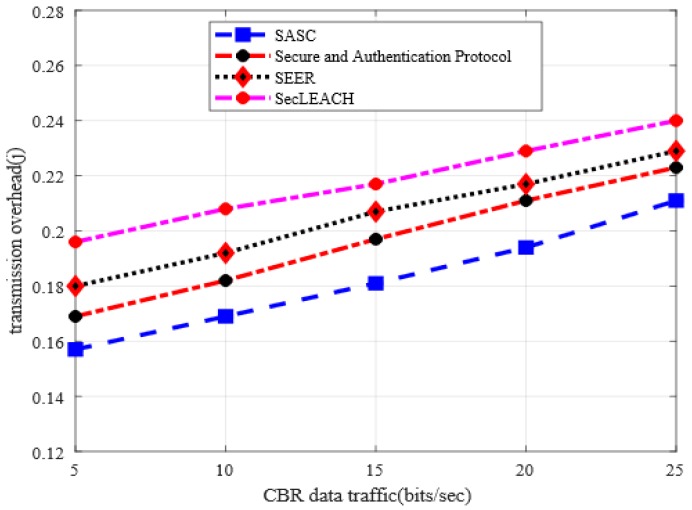
Transmission overhead in varying CBR data traffic.

Figure 10 depicts the behavior of SASC architecture in comparison with others in terms of transmission overhead under a varying number of nodes. And it is observed from the numerical results that SASC reduces the transmission overhead by an average of 15% as compared to other solutions. Similarly, the numerical analysis in Figure 11 demonstrates that SASC also improves transmission overhead by an average of 12% than the existing solutions based on varying CBR data traffic. Unlike existing solutions, the proposed technique does not impose extra communication overheads on sensor nodes. Furthermore, the existing solutions do not balance the node conditions under heavy nodes and data traffic burden. The design of the proposed SASC architecture is focused on reliability, energy-efficient and secure data routing with lightweight computation, and processing power, thus, results in decreasing transmission overhead. Furthermore, the integration of sensor-cloud infrastructure helps to provide scalable solutions with massive storage and efficient processing in a virtualized manner at minimum network overhead.

## 6. Conclusions

This paper presents a secure and authentic sensor cloud architecture, named SASC, for intelligent IoT system, which aims to guarantee data routing through secure and reliable communication links. The proposed solution offers lightweight cryptosystems to enhance security in terms of data confidentiality, integrity, and robust authentication between IoT sensors under the presence of malicious entities. The SASC architecture guides sensor nodes to send data packets on trustworthiness and authentic data forwarders while balancing the energy consumption over the network field. Additionally, integrating the cloud infrastructure to sensor networks, the proposed solution offers high quality and cost-effective communication services for IoT systems with minimal overhead. Furthermore, the SASC handles and processes all sensed data with the support of network scalability and integrity. The numerical results of SASC architecture reveal significant improvements in different network parameters as compared to other solutions. However, the proposed architecture still lacks for meeting the robust and security requirements for multi-hop communications. Furthermore, non-repudiation and playback network attacks are overlooked in the proposed SASC architecture. Therefore, we aim to improve the performance of the proposed architecture by analyzing some more misbehaving threats among cluster heads and generate an efficient and trusted end-to-end routing delivery for longer communication regions.

## Figures and Tables

**Table 1:** Default network factors.

Factor	Value
Number of malicious nodes	10
Transport layer protocol	UDP
$\;E_{elect}\;$	50 nJ/ bit
$\;E_{amp}\;$	10 nJ/bit/m2
$\;E_{fs}\;$	0.0013 pJ/bit/m4
Packet size, k	20 bits
Payload size	512 bytes
Initial energy	5 J
α, β	0.5, 0.5
Nodes transmission range	20 m
